# Data-driven super-resolution optoacoustic imaging via physically encoded signal acquisition

**DOI:** 10.21203/rs.3.rs-9315822/v1

**Published:** 2026-06-03

**Authors:** Irene Pi – Martín, Daniil Nozdriukhin, Alejandro Cebrecos, Juan José García − Garrigós, Daniel Razansky, Xose Luis Dean – Ben

**Affiliations:** 1 Instituto de Instrumentación para Imagen Molecular (i3M), CSIC – Universitat Politècnica de València, Camino de Vera S/N, 46022, Valencia, Spain; 2 Institute for Biomedical Engineering, Department of Information Technology and Electrical Engineering, ETH Zurich, Zurich, Switzerland

**Keywords:** Optoacoustic imaging, photoacoustic imaging, physically encoded acquisition, acoustic scattering, localization imaging, tracking, super-resolution imaging

## Abstract

Super-resolution optoacoustic (OA) imaging, enabled by localization and tracking of highly absorbing circulating microparticles, has overcome the longstanding resolution–depth trade-off limiting optical-contrast imaging methods. Responses recorded from individual microparticles further allow for calibrating the spatially dependent OA impulse response, which can support accurate data-driven learning of a linear forward model for tomographic reconstructions, even in complex, physically encoded acquisition schemes. We demonstrate accurate localization of microparticles by exploiting multiple ultrasound scattering to physically encode absorber positions in time-resolved OA signals. Using a model calibrated via raster scanning of a microsphere, we achieved in vivo localization and tracking of intravenously injected microparticles, enabling localization optoacoustic tomography (LOT) with over an order-of-magnitude fewer transducer elements than previously employed. Furthermore, auxiliary-transducer-assisted localization of microspheres in the bloodstream facilitated implementation of a self-calibration methodology, allowing for super-resolution imaging with a single time-resolved signal.

## Introduction

Performance of state-of-the-art optoacoustic (OA, photoacoustic) imaging systems is commonly governed by hard trade-offs between resolution, speed, and system's complexity, with most techniques using either single-element transducers for point-by-point scanning or ultrasonic arrays for parallelized detection [1–3]. High-bandwidth single-element transducers are often used for high-resolution microvascular imaging through sequential scanning of optical or acoustic foci [4–7], whereas array-based detection enables faster and deeper imaging by combining widefield illumination with simultaneous acquisition of a large number of time-resolved signals [8–11]. This fundamental dichotomy is reflected in the conventional distinction between OA microscopy (OAM, single-element-based) and OA tomography (OAT, array-based) embodiments [12].

OAT has been widely used in preclinical research and emerging clinical trials [13–16]. However, inherent complexity of the systems and excessive data rates have fostered the development of advanced sparse-acquisition OAT approaches employing a reduced number of time-resolved signals to lower overall costs [17–20] or, alternatively, accelerate the imaging frame rates [21,22]. Data-driven strategies, particularly machine and deep learning methods, have demonstrated remarkable performance in reconstructing high-quality images from only a few OA signals, outperforming conventional algorithms across multiple evaluation criteria [23–25]. These approaches effectively build a mathematical model for image reconstruction or processing by training on experimental datasets representative of expected sample variability [26]. Thereby, their performance remains strongly dependent on the similarity between training and test data, raising concerns about their generalizability to fundamentally different types of imaging objects. Alternative strategies employing signal encoding have demonstrated complementary advantages. The number of ultrasound (US) sensors required to produce OAT images has been significantly reduced by projecting acoustic wavefronts propagating through engineered structures onto time-resolved signals. Different types of structures sufficiently complex to resolve ambiguities among signals from different points have been considered, including but not limited to acoustic masks, ergodic relays, reverberant cavities, or scattering media [27–30]. The spatially-dependent OA impulse response of these systems can be experimentally determined by scanning point sources (e.g., microspheres or focused beams) [28,30], which practically represents a data driven approach to build a linear forward model for image reconstruction. This can then be used to reconstruct arbitrary samples provided linearity and tomographic coverage of waves emitted in arbitrary directions is ensured.

The recently demonstrated feasibility of super-resolution imaging represents another major breakthrough in OAT [31], overcoming the acoustic diffraction limit and enabling microvascular imaging at resolutions comparable to those achieved with high-bandwidth transducers or focused light excitation. So-called localization optoacoustic tomography (LOT) leverages microparticles with size comparable to optical or acoustic foci in high-resolution OAM [32]. These particles are sufficiently small to circulate through vascular networks, yet sufficiently absorbing to be individually detected with OAT. The sparse distribution of particles in blood enables pinpointing their positions with high precision, in a way that LOT can overcome the acoustic diffraction limit by reconstructing images with the accumulated localized positions. Recently developed microparticles, exhibiting optical absorption several orders of magnitude higher than that of red blood cells [33–38], open unique opportunities for advancing OAT systems based on physical signal encoding. On the one hand, their distribution in blood results in sparse images facilitating image reconstruction with a reduced number of sensors. On the other hand, they can act as guide stars for calibrating the spatially dependent impulse response of the system.

In this work, we demonstrate *in vivo* localization and tracking of microparticles in the bloodstream by exploiting multiple scattering of US waves to physically encode absorber positions in time-resolved OA signals. By employing an experimentally calibrated (data-driven) reconstruction framework, we establish the feasibility of LOT microvascular imaging with orders-of-magnitude fewer detection elements than those in OAT systems previously used for this method. Moreover, localization of microspheres in the bloodstream using an auxiliary transducer enabled the implementation of a self-calibration approach, allowing super-resolution imaging from a single time-resolved signal.

## Results

### Physical encoding of optoacoustic sources via acoustic scattering

**Figure 1:**
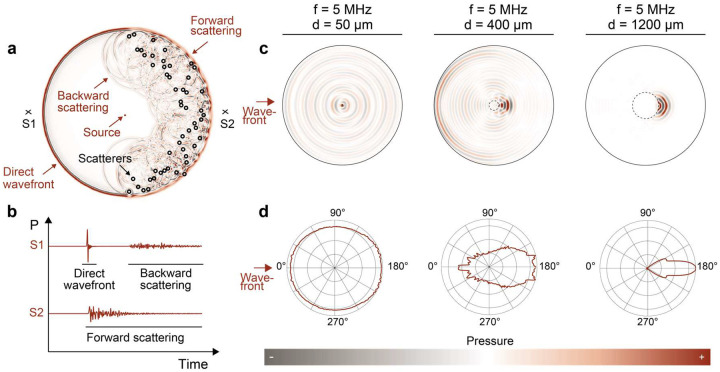
Principle of optoacoustic source encoding using acoustic scattering. (a) Simulated propagation of an ultrasound (US) wave optoacoustically generated at a point source through a uniform (scatterer-free) medium (left) and through the same medium with an embedded forest of randomly distributed acoustic scatterers (right). Simulations were performed with the k-Wave toolbox [40]. (b) Time-resolved US (pressure) signals at points S1 and S2 indicated in panel (a). The time-domain regions corresponding to direct propagation (first wavefront), forward scattering, and backward scattering are indicated. (c) Simulated wavefronts filtered with 5 MHz central frequency and 60% bandwidth for different dimensions of a hollow scatterer. (d) Directivity pattern of the amplitude of the scattered wave for the wavefronts simulated in (c).

The standard paradigm for OAT image reconstruction assumes direct, unperturbed propagation of US waves between the emission source and the receiving transducer(s), utilizing time-of-flight to determine distances and thus map source locations. In other words, OAT image reconstruction essentially consists in triangulating optical absorbers from the acquired signals. Acoustic scattering, arising from heterogeneities in the propagating medium, perturbs the wavefronts, diverting them from straight-line trajectories. Consequently, the fundamental assumption of time-of-flight-based reconstruction is violated, leading to artifacts in the images [39]. However, scattering phenomena can also be exploited to physically encode the position of optical absorbers within the acquired time-resolved OA signals. This can significantly reduce the data required for image reconstruction, enabling accurate recovery from a few, or even a single, time-resolved OA signals [30]. Numerical simulations of US propagating through a random distribution of acoustic scatterers covering a 180-degree arc illustrate the fundamental effects of scattering (Fig. 1a, see methods for details) [40]. The resulting perturbations in the signals mimic the conditions implemented in subsequent experiments.

The “ballistic” wavefront, detected in the scatterer-free region, produces a characteristic bipolar signal confined to a narrow time window (Fig. 1b), consistent with the expected response from a point source. In contrast, the signal detected on the opposite side, dominated by forward scattering, is temporally broadened, spanning a significantly longer duration. This results in a far greater number of non-zero samples in the time-domain (Fig. 1b), demonstrating that a single time-resolved signal can be informationally enriched by scattering. Notably, under conditions of strong forward scattering, the distinct ballistic component is lost, and the detected signal comprises only scattered waves. It is also important to note that backward scattering occurs, which physically encodes information on the scatterer-free side via later-arriving signal components (Fig. 1b).

The intensity ratio of forward-to-backward scattering and the overall directionality of the scattered wavefront are governed by the properties of the scatterers. Specifically, the size of the scatterer relative to the US wavelength determines whether Rayleigh or Mie scattering dominates (Fig. 1c and Suppl. Fig. 1). In the Rayleigh regime, where scatterers are much smaller than the wavelength, scattering is nearly isotropic (Fig. 1d, left). Conversely, Mie scattering, which occurs with larger scatterers, is predominantly directional and forward-focused (Fig. 1d, right). Generally, forward scattering is preferred to maximize the energy being transmitted, but in some configurations backward scattering can result in enhanced information provided the entire signals can be confined within the acquisition window (Suppl. Fig. 2).

### Optoacoustic reconstruction with an experimentally-calibrated model

**Figure 2:**
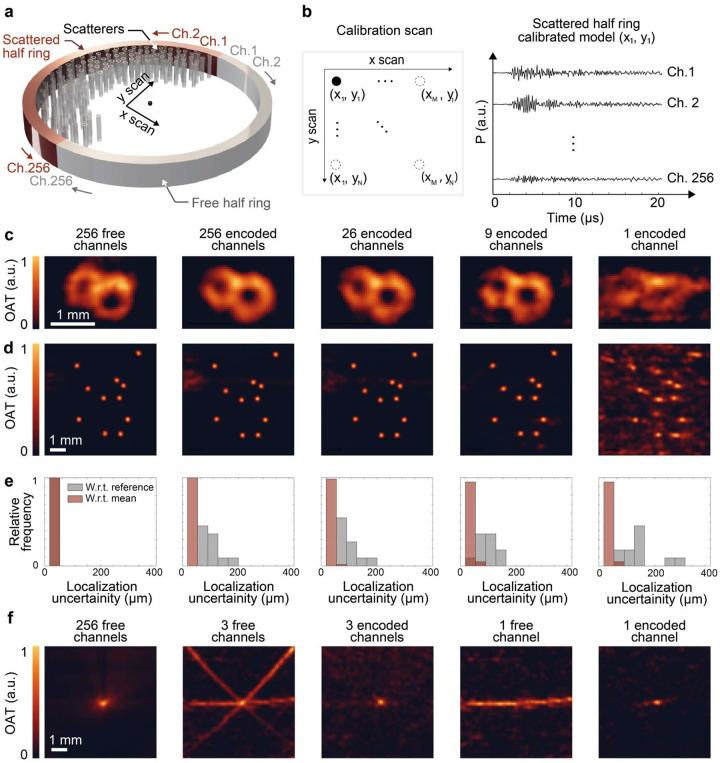
Experimentally calibrated model for optoacoustic reconstruction. (a) Lay-out of the experimental set-up indicating a microsphere being raster-scanned. (b) Schematic representation of the spatial scanning procedure and the encoded responses being recorded, serving as input data for assembling the calibrated model (columns of the model matrix). (c) Images of a phantom embedding the numeral “8” printed on a sheet of paper reconstructed by employing the direct optoacoustic (OA) signals collected by the 256 channels of the free half-ring; and by 256, 26, 6 and 1 encoded channels, respectively. (d) Images of a phantom embedding sparsely-distributed microspheres reconstructed by employing OA signals from the 256 channels of the free half-ring, and from 256, 26, 6 and 1 encoded channels, respectively. (e) Microsphere localization errors estimated as the Euclidean distances of the localized positions using encoded and direct channels for the 11 microspheres of the phantom (gray histograms) along with the Euclidean distances of the position of the localized points for 70 repeated acquisitions with respect to the average position (red histograms). (f) Image of a phantom embedding a single microsphere obtained with the OA signals from free and encoded channels, showing the effects of streak-type artefacts (arcs) in the images and the feasibility to localize individual particles.

Provided linearity is preserved, the process of OA generation, wave propagation, and detection can be formulated as a forward model that can be inverted algebraically for image reconstruction. This model-based approach has demonstrated superior performance over alternative methods and exhibits notable robustness, successfully reconstructing images from incomplete or significantly distorted data [41]. The experimental set-up employed resembles the geometry used in the previous section for the simulation of US propagation. Specifically, a full-ring array of 512 cylindrically-focused elements (channels) was used to collect time-resolved OA signals (Fig. 2a, see methods for details). A matrix of acoustic scatterers was positioned to cover half of the detection ring, enabling encoding the location of absorbers in 256 elements through forward-scattered signals (scattered half ring in Fig. 2a). The other 256 channels remain free of acoustic scatterers (free half ring in Fig. 2a) to allow the detection of waves propagating directly from source to transducer. A small microparticle (~90 μm in diameter) was scanned across the region of interest (ROI) to acquire the OA signals corresponding to a Cartesian grid of points covering such ROI, providing both encoded and direct time-resolved signals for building the forward model at each scanning point (Fig. 2b, see methods for details).

The proposed model-based methodology was evaluated by imaging representative phantoms, namely a paper substrate with a printed numeral '8' and a grid, with Tikhonov regularization being used in the inversion process (Fig. 2c and Suppl. Fig. 3, see methods for details). The reconstructed image based on 256 encoded channels is shown to exhibit sharper contours than that obtained from direct signals, arguably due to the enhanced information encoded via scattering. Comparable image quality persists with a reduction to 26 encoded elements, and the phantom structure remains distinguishable even when only 9 elements are employed. With a single encoded channel, the reconstruction exhibits pronounced distortion and decreased signal-to-noise ratio (SNR). Nonetheless, the printed structure remains discernible and its position accurately determined. Note that scattering effectively increases the angular coverage as waves initially propagating in multiple directions can be eventually redirected to a sensor at a given location, thus increasing the number of reconstruction-relevant channels (Suppl. Fig. 3). These observations highlight the robustness of the proposed encoding strategy and its potential applicability in scenarios with stringent hardware constraints, where coarse imaging or simple target localization may suffice.

The proposed methodology is particularly suitable for imaging sparse samples. This is of particular relevance for the reconstruction of sparsely-distributed microspheres flowing in blood, which can be localized and tracked along multiple frames to build super-resolution images with LOT. Microsphere localization precision and accuracy was evaluated by imaging a phantom containing eleven microspheres (Fig. 2d). All microspheres distributed across the imaging domain could be reliably identified, even with a single transducer element. Two metrics were considered to quantify localization accuracy. First, we computed the Euclidean distance from the localized positions in the reconstructed images with those obtained from direct signals of the scatterer-free half-ring, considered as a reference (Fig. 2e, gray histograms). Second, reproducibility was assessed by repeated localization of the same particle in multiple frames, with the Euclidean distance with respect to the average position being calculated (Fig. 2e, red histograms). The histograms in Fig. 2e show that, despite an increase in localization error with fewer active elements, displacements with respect to the reference position remain low (maximum 300 μm, comparable to the 100 μm step for the calibration grid). More importantly, localization reproducibility, arguably more representative of the achievable resolution, also remains low (<100 μm). Altogether, this indicates robust spatial localization under highly constrained acquisition conditions.

The enhanced localization performance achieved with encoded channels relative to scatterer-free channels is better shown by comparing the reconstructed images obtained for another phantom with a single absorbing microsphere (Fig. 2f). The microsphere is clearly discernable in the image reconstructed with 256 scatterer-free channels. However, streak artefacts in the form of arcs corresponding to individual OA signals dominate the images reconstructed with a few scatterer-free channels. These artefacts average out when considering a large number of elements, but are still prominent e.g. in the image reconstructed with 3 scatterer-free channels. On the contrary, the image reconstructed with 3 encoded channels displays a single dot enabling more accurate localization of the microsphere position. This enhanced performance achieved with encoded channels is more obvious when considering a single element. In this case, the position of the microsphere cannot be determined in the single arc dominating the image reconstructed with a scatterer-free channel, while localization of a single peak is still possible when using an encoded channel for image reconstruction.

### *In vivo* imaging of the murine cerebral microvasculature assisted with flowing microparticles

**Figure 3:**
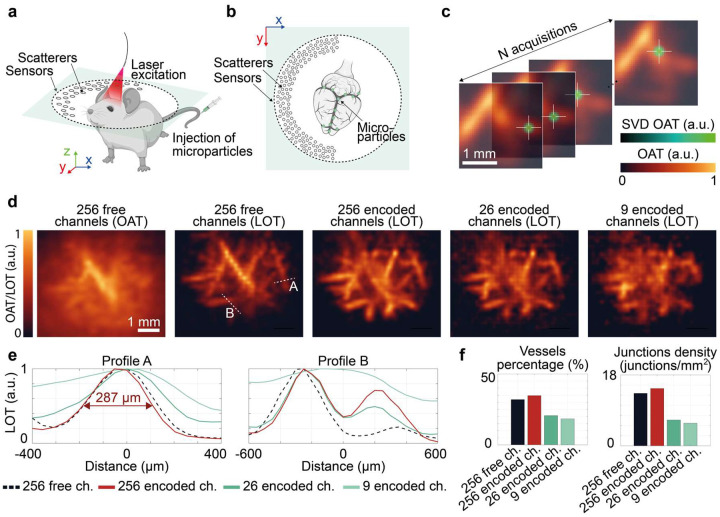
*In vivo* imaging of the murine cerebral microvasculature with the experimentally calibrated model. (a) Schematic representation of the experimental workflow in a mouse model based on intravenous administration of microparticles for localization optoacoustic tomography (LOT). (b) Cross-sectional view of the horizontal imaging plane, with intravenously-injected microparticles circulating through the cerebral vasculature. (c) Sequence of optoacoustic tomography (OAT) images along with the corresponding singular value decomposition (SVD)-based filtered sequence enabling differentiating individual flowing microparticles. (d) OAT image reconstructed with the calibrated model considering the 256 channels of the free half ring along with the LOT images obtained with the same channels as well as with 256, 26, and 9 encoded channels, respectively. (e) Image profiles for the LOT images indicated in panel (d). (f) Vessel percentage area and junctions density for the LOT images shown in panel (d) [42].

Localization of individual particles provides a means of building a LOT image of vascular structures with the accumulated localized positions. The feasibility of particle localization with the physical encoding method based on acoustic scatterers was validated *in vivo* using highly absorbing microparticles flowing through the cerebral vasculature. Specifically, coreless polyelectrolyte microcapsules (MCs) containing a high concentration of indocyanine green (ICG) as light absorbing agent were intravenously (i.v.) injected in mice [34] (see methods for details). The mouse was immobilized relative to the transducer array to enable imaging of a horizontal section of the murine cerebral cortex (Fig. 3a–b, see methods for details). Singular value decomposition (SVD)-based filtering of the sequence of acquired images enables distinguishing flowing particles causing image fluctuations from the highly absorbing static blood background (Fig. 3c). The LOT image reconstructed with the 256 scatterer-free channels is shown to outperform the standard OAT image reconstructed with the calibrated model in terms of visible microvascular networks (Fig. 3d). Even when using a theoretical model with smaller pixel size (36.1 μm), only a few vascular networks are visible (Suppl. Fig. 4). A similar LOT image of the microvasculature is achieved via image reconstruction with 256 encoded channels (Fig. 3d). Progressive reduction in the number of active detection elements decreases SNR in the LOT image. However, vascular morphology remains largely preserved with 26 channels, and overall anatomical features are still identifiable with just 9 channels (Fig. 3d). The comparable imaging performance achieved with 256 free channels and with a reduced number of encoded channels is further supported by the image profiles of selected vessels (Fig. 3e). Indeed, blood vessel resolution remains largely preserved, with significant deterioration observed only when the number of encoded channels is decreased by more than an order of magnitude. Quantitative analysis of the vascular networks further revealed that several morphological and spatial parameters exhibit improved values with 256 encoded channels compared to 256 free channels (Fig. 3f and Suppl. Fig. 5, see methods for details) [42], consistent with the observations in the phantom experiments.

### Super-resolution imaging with a self-calibrated model

**Figure 4.**
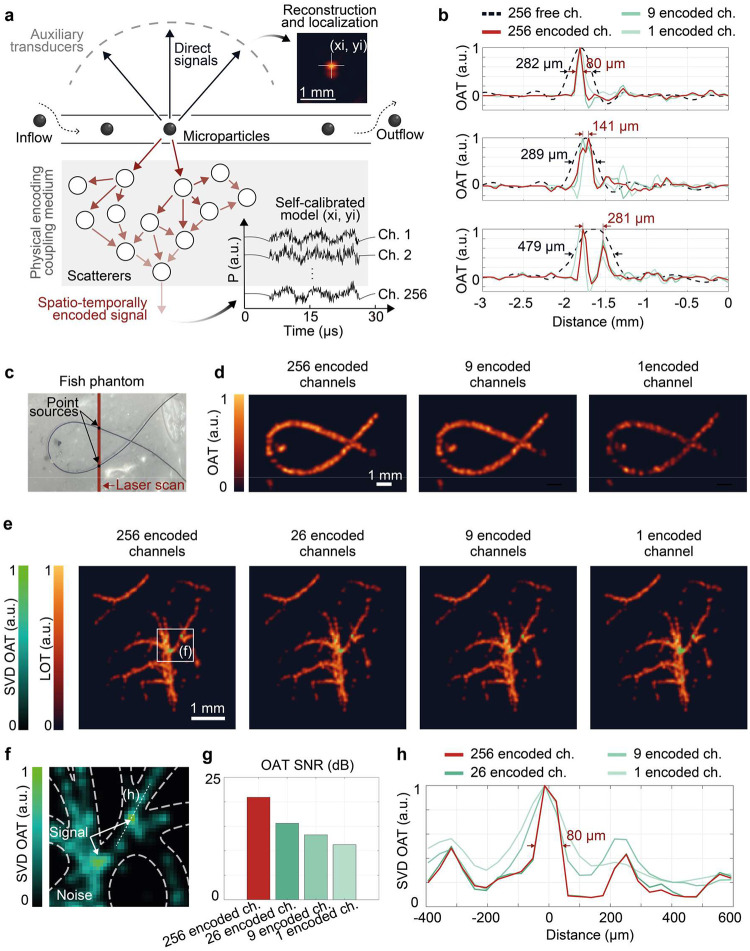
Self-calibrated reconstruction framework for super-resolution imaging. (a) Schematic representation of the self-calibration methodology based on flowing microparticles. (b) One-dimensional reconstructed profiles for microparticles flowing through a tubing obtained with standard optoacoustic tomography (OAT) reconstruction and with the self-calibrated model based on encoded channels. (c) Two-dimensional agar phantom embedding a black thread and a microsphere forming the shape of a fish, with a line-shaped laser beam scanned across the phantom to create point sources. (d) Reconstructed images of the phantom obtained with the self-calibrated model for 256, 9, and 1 encoded channels, respectively. (e) Reconstructed images from the singular value decomposition (SVD)-filtered sequence using the self-calibrated model (SVD OAT) along with the localization optoacoustic tomography (LOT) images formed with the accumulated localized positions. (f) Zoom-in of the SVD-filtered OAT image for the region indicated in panel (e). (g) Signal-to-noise ratio (SNR) of the SVD-filtered OAT images estimated as the peak signal intensity normalized with the standard deviation of non-zero values in the selected region shown in panel (e). (h) Image profiles of the SVD-filtered OAT images indicated in panel (f).

The calibration procedure described above relies on Cartesian scanning of a point source, which generally becomes prohibitively time-consuming when high spatial resolution is required. The long duration of the scan may further lead to speed of sound variations associated with temperature instability. Moreover, minor positional shifts of acoustic scatterers or other hardware components may occur when the calibration phantom is replaced with the sample of interest, which additionally exhibits different acoustic properties and structural heterogeneity. Model uncertainties associated with these effects can be mitigated by employing a self-calibration procedure based on dynamic point sources within the sample of interest. The positions of such sources can be accurately localized in specific frames with auxiliary transducers, such as the elements of the free half ring used for the experiments, with signals from encoded channels corresponding to the localized position(s) being simultaneously collected (Fig. 4a, see methods for details).

The performance of the proposed self-calibration method was first evaluated using microparticles flowing within a tubing corresponding to a one-dimensional structure (Fig. 4a–b, see methods for details). Accordingly, a one-dimensional model was built, allowing reconstruction of the longitudinal profile along the tubing. Notably, the profile corresponding to an individual microsphere reconstructed with the self-calibrated model is significantly narrower than that obtained with standard reconstruction, even when only a single channel is employed (Fig. 4b, top). Also, microparticles can still be accurately localized with a reduced number of encoded channels (Suppl. Fig. 6). Importantly, the self-calibrated model enabled distinguishing individual microparticles in close proximity as independent emitters, thereby improving spatial resolution [43] beyond the capability of standard OAT (Fig. 4b, middle and bottom), effectively breaking the acoustic diffraction barrier that otherwise limits the resolution of the system.

The method was further experimentally validated in a two-dimensional scenario using a black thread and a microsphere embedded in an agar phantom to form the shape of a fish, with point sources created by scanning a line-shaped beam across the sample (Fig. 4c, see methods for details). Following the calibration scan, the cylindrical lens used for laser beamforming was removed, and the phantom was uniformly illuminated to acquire single-shot, wide-field OAT images with 256, 9, and 1 encoded channels, respectively (Fig. 4d). The reconstructed images retain the resolution and contrast of the image corresponding to the accumulated localized positions, as non-zero values are exclusively produced at these points. As expected, the distribution of image intensities for non-zero pixel values, corresponding to the localized points, exhibits higher variations when decreasing the number of channels (Suppl. Fig. 7). However, a representative image can still be produced with a single channel for wide-field illumination, indicating the robustness of the method.

*In vivo* imaging of the murine cortical vasculature further demonstrated the advantages of the self-calibration methodology. Specifically, a sequence of OAT images (6 minutes at 100 frames per second) was acquired following i.v. injection of a bolus of MCs (see methods for details). The self-calibrated model was built with the first 1800 SVD-filtered frames of the acquired dataset, which was then used to reconstruct the subsequent 1800 frames of the filtered sequence. Individual dots are clearly distinguishable in the reconstructed images (Fig. 4e–f), with sufficient SNR to enable localization of flowing MCs even using a single encoded channel (Fig. 4g). The LOT images built with the accumulated localized positions for a relatively low number of frames preserve the super-resolution capacity established by the points localized in the self-calibration procedure, even for a single encoded channel (Suppl. Fig. 8), while faster acquisitions are facilitated with the reduced data rate. Notably, particles can also be more accurately resolved along the vessel direction after self-calibration (Fig. 4h), which facilitates tracking of highly absorbing circulating particles or cells.

## Discussion and conclusions

The propagation of US waves through a complex distribution of acoustic scatterers provides an effective means of encoding the location of optical absorbers within time-resolved OA signals of relatively long duration. The broadband nature of OA emissions results in varying acoustic scattering behavior (Rayleigh or Mie) across the frequency spectrum, mainly governed by the ratio of scatterer size to acoustic wavelength [44]. High-frequency components, essential for resolving small microparticles in blood, typically undergo Mie scattering (acoustic wavelength < scatterer dimensions), which is primarily forward directed and thus results in efficient propagation of US waves towards the transducer. This further preferentially filters high over lower frequencies subject to more isotropic Rayleigh scattering.

Despite the general preference for forward scattering, the simulated data for an array of cylindrically-focused transducers reveals that backward-scattered waves also carry substantial information for reconstruction. Resonant cavities, formed by one or several sensing elements and solid boundaries around the sample, enable image reconstruction by capitalizing on both forward-scattered waves and multiple reverberations. The use of reverberant cavities for single-element OA imaging has long been theoretically proposed and more recently experimentally validated [29,45]. The incorporation of scattering surfaces or elements within the cavity can further enhance information encoding, which is facilitated by the inherently long duration of reverberant signals. However, a key practical constraint is the duration of the digitized signal, which is limited by data acquisition electronics and the PRF of the laser. High-speed imaging (>1 kHz) requires sub-millisecond acquisitions [21,46]. Forward-scattering-based encoding results in time-resolved signals with shorter duration than those involving extensive reverberation and avoids signal overlap between laser pulses. This strategy prioritizes less prolonged waveforms over the full complexity of reverberation.

The imaging setup, based on a full-ring acquisition geometry, was designed to validate the performance of acoustic-scattering-mediated imaging of sparse samples, particularly microparticles enabling super-resolution OA imaging (LOT) [31,47]. The symmetry of the system enables direct comparison and validation of results. On one side, 256 elements record unscattered waves in the scatterer-free side, serving as a reference for full-data image reconstruction and for establishing ground-truth LOT images. The complementary 256 elements on the opposite (“scattering”) side are used to assess how wave propagation through scatterers provides positional encoding, thereby reducing the number of signals required for accurate LOT imaging. In the multiple-scattering regime, the diffuse approximation allows the time duration of the scattered wavefield to be estimated by the Thouless time, L^2^/D, where L is the medium thickness and D the diffusion coefficient [48]. For a given sampling frequency, defined by the maximum signal frequency, the duration of the acoustic-scattering-encoded signal determines the number of independent values recorded per signal acquisition. For instance, propagation through a scattering medium increases the number of nonzero entries in the digitized, time-resolved OA signal corresponding to a point source. This increase of nonzero values, in turn, reduces the condition number of the reconstruction matrix (Suppl. Fig. 3c), thereby facilitating the recovery of sparse absorbers from fewer signals.

The proposed methodology relies on a model-based reconstruction framework to map the distribution of optical absorbers to the acquired time-resolved signals. This approach, which generally outperforms analytical techniques like filtered back-projection [41], involves building a forward model that can incorporate acoustic heterogeneities, attenuation, the spatially dependent impulse response of US sensors, and other relevant factors related to propagation and transduction of pressure waves [49,50]. The model is generally derived theoretically from the OA wave equation and the known geometry of the detection system. Alternatively, it can be built experimentally by recording the response of the system to a point-like absorber scanned throughout the region of interest [51]. Notably, under the linearity conditions inherent to low-intensity OA waves, the signal from any arbitrary absorption distribution is a linear superposition of these individual point-source responses, weighted by the absorption strength at each point. In this work, we employed the latter, experimental approach to calibrate our model. This data-driven strategy is generally applicable to any scattering environment and is often more accurate than theoretical modeling, as it inherently accounts for practical tolerances, uncertainties in scatterers positions, and other experimental imperfections.

Data-driven methods have revolutionized biomedical imaging and other fields. Supervised deep learning, based on convolutional neural networks (CNNs) trained with paired input and output (“ground truth”) images, enables accurate biomedical image analysis by learning from labeled data to classify relevant features, remove artefacts, or enhance contrast or resolution [24,52]. The self-calibration procedure introduced herein, where images of several moving sources are used to build a model, is conceptually analogous to supervised deep learning methods. Both paradigms are fundamentally data-driven, learning a mapping function from a set of training examples, in this case the measured signals and their corresponding known source locations. This allows the model to implicitly learn and compensate for the complex acoustic environment, including the effects of scatterers and transducer responses. The primary difference lies in the model architecture. While deep learning employs complex, hierarchical CNNs to approximate non-linear functions, the proposed approach leverages the inherent linearity of the OA forward model. Consequently, the solution simplifies to a single transformation matrix, which is both computationally efficient and inherently interpretable.

In conclusion, we demonstrated an efficient LOT imaging approach that leverages physically encoded signals from multiply scattered ultrasound to achieve super-resolution imaging with drastically reduced data acquisition. We introduced a data-driven calibration methodology to build a linear reconstruction model, which we then applied to achieve *in vivo* microparticle localization and tracking. By further using auxiliary transducers for microparticle localization, we proposed a self-calibration scheme capable of super-resolution imaging from a single time-resolved signal, validating a new paradigm for simplified, high-performance OA imaging.

## Methods

### Numerical simulations

Two-dimensional numerical simulations were performed using the k-Wave pseudo-spectral time-domain toolbox [40]. Specifically, a 512-element ring transducer (77.8 mm in diameter) was modelled over an 81×81 mm^2^ domain with 95.67 μm pixel size. Air scatterers were randomly distributed on one side of the ring to spatiotemporally encode the detected signals. The same framework was used to build a theoretical numerical model for a central ROI of 3.16×3.16 mm^2^ with 95.67 μm spatial resolution. This model was used for the reconstruction of images from simulated signals (Suppl. Fig. 2).

### Acoustic scatterers

Borosilicate glass capillaries with 1.17 and 1.5 mm inner and outer diameters, respectively (Harvard Apparatus, Holliston, USA) were sealed with epoxy resin (Deluxe Materials Ltd., UK) to confine the internal air volume. A polylactic acid (PLA) holder, fabricated by fused deposition modelling 3D printing (Bambu Lab, Shenzhen, China), was used to arrange the capillaries in a matrix configuration. The holder–capillary assembly was mounted onto a custom-made full-ring transducer array described in the next section.

### Full-ring-based experimental system

A custom-made full-ring transducer array with 512 cylindrically-focused elements (Imasonics, Voray-sur-l'Ognon, France) was used in the experiments, providing 360° coverage, a central frequency of 5 MHz, and a detection bandwidth exceeding 80% [53]. An acoustic scatterer matrix covered 180° degrees, enabling spatiotemporal encoding of 256 elements via forward-scattered signals, while the remaining 256 elements were left without scatterers in the propagating path for direct (“clean”) detection.

### Optoacoustic imaging

For OA imaging, optical excitation was provided by a fiber-bundle-coupled laser source (InnoLas Laser GmbH, Krailling Germany) operating at 100 Hz and delivering ~35 mJ pulses at 800 nm. The output beam guided through a custom-made fiber bundle (CeramOptec, Bonn, Germany) was aligned vertically to illuminate the center of the ring. Phantoms were placed at the ring center and, during *in vivo* experiments, the mouse head was immobilized with a stereotactic frame to ensure alignment of the cerebral imaging plane with the focal plane of the ring.

### Light-absorbing microparticles

Super-resolution OA imaging was achieved with microparticles providing orders of magnitude larger light absorption than red blood cells. Specifically, shell-loaded polyelectrolyte microcapsules (MCs) were synthesized following an established protocol [34]. ICG-loaded CaCO_3_ cores were prepared by coprecipitation of CaCl_2_ and Na_2_CO_3_ in the presence of ICG, followed by centrifugation and washing. Layer-by-layer assembly was then performed by alternating PDDA and PSS adsorption, yielding a multilayered structure: CaCO_3_ / [PDDA/PSS]_2_ / [PDDA/FeNP]_4_ / [PDDA/ICG]_4_ / [PDDA/PSS]_2_. The vaterite cores were eventually dissolved with HCl, and the resulting hollow MCs were purified by repeated washing. Owing to their high optical absorbance, these MCs were specifically designed to enable detection as individual contrast agents during *in vivo* experiments.

### Scanning-based model calibration

For phantom experiments (Fig. 2), a black paramagnetic polyethylene microsphere with a diameter of ~90 μm (Cospheric, Santa Barbara, USA) was raster-scanned across the region of interest (ROI) using a motorized positioning system with 100 μm step size (IAI, Shizuoka Prefecture, Japan), covering an area of 7.2×7.2 mm^2^. At each step, OA responses at 720 nm were recorded and averaged 200 times across all transducer elements, providing the system’s impulse response at each spatial location. These responses were compiled into a model matrix, associating each point in the ROI with its corresponding transducer response. The same procedure was performed for the *in vivo* experiments using a ~200 μm diameter microsphere and a scanning step size of 200 μm (Fig. 3).

### Self-calibrated model

A self-calibration procedure was implemented in two stages: localization of sources within the ROI, and assignment of the corresponding impulse responses to spatial coordinates. In contrast to scanning-based calibration methods requiring controlled source positioning and model-based fitting, this approach enables freely moving sources within the imaging domain. The part of the array with no acoustic scatterers detected the direct signals, and source positions were reconstructed from time-of-flight estimation. These positions were then used to map the impulse responses acquired through spatiotemporal encoding for the part of the array with acoustic scatterers.

### Phantom experiments

Phantoms were fabricated by embedding a black numeral “8” (Fig. 2c) and a grid (Suppl. Fig. 2) printed on a sheet of paper, a black thread (Fig. 4c–d), and 200 μm polystyrene microspheres (Cospheric, USA) (Fig. 2d) within agar cylinders. The phantoms were imaged using direct signals from 256 scatterer-free elements and spatiotemporally encoded signals from complementary subsets of elements positioned behind the scattering medium. Reconstructions were performed using model-based approaches with Tikhonov regularization, and the inverse problem was solved with the LSQR algorithm truncated after 100 iterations.

### *In vivo* animal experiments

*In vivo* experiments were performed on mice to evaluate the capability of the system for LOT. The animals were maintained under isoflurane anesthesia throughout the procedure. A bolus of 100 μL of FeNP/ICG-loaded polyelectrolyte MCs was administered intravenously via tail vein injection approximately 20 s after acquisition of a baseline image. The mouse brain was imaged transcranially (skull intact). Animal experiments were conducted following the Swiss Federal Act on Animal Protection and were approved by the Cantonal Veterinary Office Zurich. The mice were housed in individually ventilated, temperature-controlled cages under a 12-hour dark/light cycle, with pelleted food (3437PXL15, Cargill) and water available *ad libitum*. For imaging, the animals were anesthetized with isoflurane (5% v/v for induction and 1.5% v/v for maintenance, Abbott, Cham, Switzerland) delivered in an oxygen/air mixture (100/400 mL/min).

## Supplementary Material

Supplementary Files

This is a list of supplementary files associated with this preprint. Click to download.

- Supplementary.pdf

## Data Availability

The main data supporting the findings of this study are available within the main text or Supplementary information. The raw datasets are available for research purposes from the corresponding author upon request.

## Code availability

Data analysis was performed with custom MATLAB codes which are available for research purposes from the corresponding author upon request.
